# Impact of Oral Health Status on Postoperative Complications and Functional Recovery After Cardiovascular Surgery

**DOI:** 10.1016/j.cjco.2020.10.007

**Published:** 2020-10-13

**Authors:** Masato Ogawa, Seimi Satomi-Kobayashi, Naofumi Yoshida, Yasunori Tsuboi, Kodai Komaki, Nagisa Nanba, Kazuhiro P. Izawa, Takeshi Inoue, Yoshitada Sakai, Masaya Akashi, Ken-ichi Hirata, Kenji Okada

**Affiliations:** a Division of Rehabilitation Medicine, Kobe University Hospital, Kobe, Japan; b Department of Public Health, Kobe University Graduate School of Health Sciences, Kobe, Japan; c Division of Cardiovascular Medicine, Department of Internal Medicine, Kobe University Graduate School of Medicine, Kobe, Japan; d Department of Oral and Maxillofacial Surgery, Kobe University Graduate School of Medicine, Kobe, Japan; e Division of Cardiovascular Surgery, Department of Surgery, Kobe University Graduate School of Medicine, Kobe, Japan; f Division of Rehabilitation Medicine, Kobe University Graduate School of Medicine, Kobe, Japan

## Abstract

**Background:**

Poor oral health status can lead to a deteriorated level of general health and is common among patients undergoing cardiovascular surgery. However, the effect of oral health status on postoperative outcomes in cardiovascular surgery patients remains unclear. Thus, we investigated the effect of preoperative oral health status on postoperative complications and functional recovery after cardiovascular surgery.

**Methods:**

This single-centre retrospective cohort study included 884 inpatients undergoing elective cardiovascular surgery. Oral health status was assessed based on the number of remaining teeth, use of dentures, occlusal support, and periodontal status. We investigated postoperative complications related to surgery and postoperative functional recovery by measuring the reacquisition of walking ability, activities of daily living, and length of postoperative hospital stay.

**Results:**

In this cohort (age 66.9 ± 13.4 years), the mean number of remaining teeth was 18.7 ± 9.4. Patients were grouped based on tertiles of the data distribution of remaining teeth: ≥ 20 teeth (470 patients); 10-19 teeth (137 patients); < 10 teeth (185 patients). The number of missing teeth was associated with age (*P* < 0.001). The prevalence of postoperative pneumonia and reintubation after surgery was 3.2% and 2.5%, respectively, which was significantly higher in patients with severe tooth loss (*P* < 0.05 for both). After adjusting for age and other confounding factors, the number of remaining teeth was a statistically significant predictor of functional recovery (*P* < 0.05).

**Conclusions:**

Preoperative oral health status was related to postoperative respiratory complications and independently associated with functional recovery. Preoperative oral intervention may improve functional recovery after cardiovascular surgery.

## Résumé

**Contexte:**

Une mauvaise santé buccodentaire peut avoir des répercussions néfastes sur la santé générale et est fréquemment observée chez les patients subissant une intervention chirurgicale cardiovasculaire. Cependant, l’effet de l’état de santé buccodentaire sur les issues postopératoires chez les patients ayant subi une telle intervention est mal connu. Nous avons donc étudié l’effet de l’état de santé buccodentaire avant l’intervention sur les complications postopératoires et le rétablissement fonctionnel après une intervention chirurgicale cardiovasculaire.

**Méthodologie:**

Cette étude de cohorte rétrospective menée dans un seul centre réunissait 884 patients subissant une intervention chirurgicale cardiovasculaire non urgente. L’état de santé buccodentaire des patients a été évalué en fonction du nombre de dents restantes, du recours à des prothèses dentaires, de la présence d’appuis occlusaux et de la santé parodontale. Nous avons examiné les cas de complications postopératoires liées à l’intervention chirurgicale ainsi que le rétablissement fonctionnel après l’intervention en évaluant la réacquisition de la capacité de marcher, la capacité d’accomplir les activités de la vie quotidienne et la durée du séjour à l’hôpital après l’intervention.

**Résultats:**

Dans cette cohorte (âge moyen : 66,9 ± 13,4 ans), les sujets avaient en moyenne 18,7 ± 9,4 dents restantes. Les patients ont été répartis en trois groupes en fonction du nombre de dents restantes : ≥ 20 dents (470 patients); de 10 à 19 dents (137 patients); < 10 dents (185 patients). Il y avait un lien entre le nombre de dents manquantes et l’âge (*p <* 0,001). La prévalence des cas de pneumonie postopératoire et de réintubation après l’intervention s’établissait à 3,2 % et à 2,5 %, respectivement, et était significativement plus élevée chez les patients à qui il restait très peu de dents (*p* < 0,05 dans les deux cas). Après correction pour tenir compte de l’âge et d’autres facteurs de confusion, le nombre de dents restantes s’est révélé être un facteur de prédiction statistiquement significatif du rétablissement fonctionnel (*p* < 0,05).

**Conclusions:**

L’état de santé buccodentaire avant l’intervention chirurgicale était lié à des complications respiratoires postopératoires et associé de manière indépendante au rétablissement fonctionnel. Des soins buccodentaires préopératoires pourraient améliorer le rétablissement fonctionnel après une intervention chirurgicale cardiovasculaire.

Poor oral health status is common among older hospitalized patients. More than 90% of patients in acute care hospitals reportedly experience impaired oral health.^1^ In particular, patients with cardiovascular disease (CVD) have a high prevalence of poor oral health status due to comorbidities associated with periodontal disease.^2^ Furthermore, cardiovascular surgery further impairs oral health status due to prolonged perioperative endotracheal intubation.^3^^,^^4^

Recently, decreased oral function has been referred to as “oral frailty.”^5^ Oral frailty is defined as a series of phenomena and processes that lead to changes in various oral conditions associated with aging, which is accompanied by decreased interest in oral health.^5^ Increased oral frailty leads to deterioration of physical and mental function and the development of physical frailty.^5^ Because oral frailty could lead to frailty progression via malnutrition or chronic inflammation, detailed countermeasures are required.^6^ Oral frailty can be reversed by various interventions according to each frailty level. Thus, awareness of oral frailty could prevent frailty progression and break the -vicious cycle that could lead to further increase of frailty.

Furthermore, preoperative frailty in cardiovascular surgery patients is a known independent predictor of mortality or major morbidity and decreased functional recovery in the rehabilitation setting.^7^, ^8^, ^9^ Perioperative oral intervention and optimal strategies for such intervention are recommended; however, the effect of oral health status on postoperative outcomes in cardiovascular surgery patients is rarely considered.^10^ Importantly, postoperative functional recovery or activity of daily living (ADL) is of crucial importance because inadequate functional recovery or ADL at discharge predicts adverse cardiac events over the next year.^11^^,^^12^ Therefore, we investigated the relationship between preoperative oral health status and functional recovery or postoperative outcomes after cardiovascular surgery.

## Methods

### Study population

**Figure 1:**
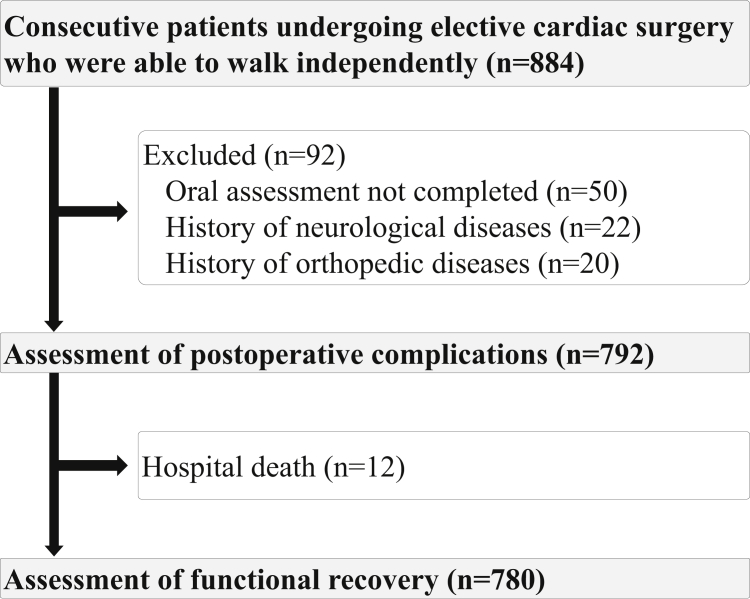
Flowchart of patients in the study.

This retrospective cohort study was conducted from May 2014 to December 2018 at a single university hospital located in an urban area of Japan. We enrolled 884 consecutive inpatients who underwent elective cardiovascular surgery and were able to walk independently with or without a walking aid before surgery. Patients who had neurologic or severe orthopedic disease in whom postoperative rehabilitation was not achieved, and those for whom oral function could not be evaluated before surgery, were excluded (Fig. 1). This study complied with the principles of the Declaration of Helsinki regarding investigations in human subjects and was approved by the Kobe University Institutional Review Board (approval no. 190064). Because of the retrospective study design, we used the opt-out method for obtaining consent.

### Patients’ clinical characteristics

We evaluated the baseline characteristics, including sociodemographic data, laboratory data, comorbidities, medications, echocardiographic data, and operative risk scores, such as the **Euro**pean **S**ystem for **C**ardiac **O**perative **R**isk **E**valuation (EuroSCORE) II.^13^ Nutritional status at baseline was assessed using the Mini Nutritional Assessment Short-Form (MNA-SF).^14^ Physical function was assessed using the Short Physical Performance Battery (SPPB), handgrip strength, and muscle mass.^15^ Muscle mass was estimated according to the cross-sectional areas of the left and right psoas muscles at the level of the transverse process of L3 divided by the height squared and diagnosed as previously described.^16^ Postoperative clinical variables included hospital mortality and postoperative complications associated with surgery, such as postoperative pneumonia and renal failure. Postoperative pneumonia was defined as a clear diagnosis of pneumonia on computed tomography or suspected pneumonia on computed tomography with a fever of least 38 °C and blood leukocytosis of least 11,000/μL, with no other site of infectious disease.^17^

### Assessment of oral status and perioperative oral interventions

Participants underwent oral examinations by a trained dentist and dental hygienist within 1 week before surgery. All patients underwent routine panoramic radiography. The oral examination consisted of counting the remaining and functional teeth, defined as the sum of the numbers of natural teeth, implant-supported artificial teeth, and fixed and removable prostheses. Severely decayed teeth and teeth stumps were not considered functional teeth. All dental diseases (caries, apical periodontitis, marginal periodontitis, and impacted wisdom teeth), unfitted dental restorations (inlay, crown, or bridge), and denture use were evaluated. Dentists carefully evaluated loose teeth at high risk of avulsion during intubation and planned the timing of prophylactic dental extraction, as necessary. Teeth with marginal periodontitis with severe mobility were candidates for prophylactic dental extraction. When patients required immediate surgery, invasive tooth extraction and scaling were generally avoided, and non-preservable teeth were conservatively treated, even if their condition warranted extraction.

All patients underwent professional mechanical tooth cleaning by a trained dental hygienist. Based on the number of decayed (D), missing (M), and filled (F) teeth (T), the DMF-T index was determined.^18^ The DMF-T index enables conclusions regarding the caries experience of an individual. Occlusal support was classified according to the Eichner index,^19^ a widely used index to assess occlusal support status based on existing natural teeth contact. We divided patients into 3 groups—Group A: natural dentition with adequate function; Group B: partially or fully edentulous but maintaining functional occlusion with dentures in either or both jaws; and Group C: functionally inadequate occlusion with no dentures. The Community Periodontal Index of Treatment Needs (CPITN) was used to assess periodontal status.^20^ The CPITN measures the following: no treatment needs (CPI 0), bleeding gingiva on gentle probing (CPI 1), presence of supra- or sub-gingival calculus or other plaque-retentive factors (CPI 2), 4- or 5-mm-deep periodontal pockets (CPI 3), and 6-mm or deeper periodontal pockets (CPI 4). Preoperative oral health assessment by dentist and dental hygienist took approximately 30 minutes to 1 hour, including professional mechanical tooth cleaning.

### Functional recovery

All patients underwent postoperative rehabilitation from the day following surgery, according to the Japanese Circulation Society guidelines for rehabilitation in patients with CVD.^21^ We investigated postoperative reacquisition of walking ability, postoperative ADL, and length of postoperative hospital stay as indicators of postoperative functional recovery. Reacquisition of walking ability was defined as the ability to walk 100 m without supervision by medical staff, regardless of the duration or whether a brace or walking aid was used.^8^^,^^22^ To evaluate ADL, the Barthel index was measured at hospital discharge.^23^

### Statistical analysis

We conducted statistical analyses after confirming that the data were normally distributed using the Shapiro-Wilk test. To explore the association between oral status and clinical characteristics, patients were stratified into 3 groups according to the baseline number of remaining teeth (≥ 20, 10-19, < 10), as previously described.^24^^,^^25^ Continuous variables were expressed as mean ± standard deviation and compared using 1-way analysis of variance. Categorical variables are expressed as numbers (percentages), and Fisher’s exact test was used to evaluate the differences. Post-hoc analyses were performed using the Bonferroni correction for multiple comparisons, when appropriate. Multiple regression analyses were performed for postoperative functional recovery measured by postoperative reacquisition of walking ability, postoperative ADL, and length of postoperative hospital stay as dependent variables, and number of remaining teeth and other clinical characteristics as independent variables. Factors theoretically related to postoperative functional recovery were included as confounding factors, such as age, sex, body mass index, MNA-SF, SPPB, EuroSCORE II, postoperative complications, type of surgery, New York Heart Association class, chronic kidney disease (CKD), and smoking status. The unstandardized regression coefficient (β) was calculated in the multiple regression analyses. *P*-values <0.05 were considered statistically significant. Statistical analyses were performed using R, version 2.8.1 (The R Foundation for Statistical Computing, Vienna, Austria).

## Results

### Clinical characteristics

Of the 884 patients, 92 were excluded because postoperative rehabilitation could not be achieved due to neurologic or orthopedic disease in 42 patients, and oral status could not be assessed in 50 patients. Thus, 792 patients with a mean age of 66.9 ± 13.1 years were included (Fig. 1). The number of remaining teeth was associated with aging (*P* < 0.001; Table 1). Participants with comorbidities such as hypertension, CKD, chronic obstructive pulmonary disease, and diabetes had significantly more missing teeth than those without comorbidities (*P* < 0.05 for all). Patients with a low MNA-SF score had significantly fewer remaining teeth (*P* < 0.001). Furthermore, preoperative physical function measures such as handgrip strength, gait speed, and SPPB score were lower in patients with < 10 remaining teeth than in those with ≥ 20 remaining teeth (*P* < 0.05 for all). The mean number of remaining teeth was 18.7 ± 9.9 (Table 2). The prevalence of denture use was 34.8% overall, and the ratio of denture use was higher in patients with fewer remaining teeth. The periodontal status assessed by CPITN was poorer in the group with fewer remaining teeth. Deeper periodontal pockets, indicated by a CPITN score ≥4, were observed in 137 patients (17.3%). The proportion of patients with a CPITN score ≥4 was highest (32.4%) in patients with 10-19 teeth, indicating that periodontitis was more prevalent among them than among patients with <10 teeth. The prevalence of occlusal support stratified by the Eichner index was 39.3%, 35.6%, and 25.1% for Class A, B, and C, respectively. Occlusal support was better with a higher number of teeth. A total of 96 patients (12.1%) underwent prophylactic dental extractions. Patients with 10-19 teeth required the greatest proportion of tooth extractions before surgery (*P* < 0.001).

**Table 1:** Baseline clinical characteristics of the patients according to the number of remaining teeth

Variable	Number of teeth			*P*
	≥ 20	10-19	< 10	
Number	470	137	185	
Age, y	62.50 ± 13.95∗^,^†	70.94 ± 8.60†	74.11 ± 8.47	< 0.001
Sex, female	325 (69.1)∗	78 (56.9)	121 (65.4)	0.028
BMI, kg/m^2^	23.45 ± 4.47	22.62 ± 4.25	23.06 ± 4.07	0.125
Lab data				
Albumin, g/dL	4.05 ± 1.87	3.82 ± 0.73	4.06 ± 2.83	0.461
BNP, pg/mL	210.32 ± 177.36	247.58 ± 249.86	347.48 ± 210.86	0.284
Hemoglobin, g/dL	12.93 ± 2.24∗^,^†	12.13 ± 2.60	12.40 ± 2.48	0.001
eGFR, mL/min per 1.73 m^2^	60.50 ± 25.81∗^,^†	51.45 ± 23.48	48.98 ± 25.67	< 0.001
CRP, mg/dL	0.42 ± 1.01	0.46 ± 1.11	0.50 ± 0.95	0.679
Comorbidity				
Diabetes	75 (16.0)†	23 (16.8)	52 (28.1)	0.007
COPD	162 (34.5)†	68 (49.6)	95 (51.4)	< 0.001
Hypertension	286 (60.9)†	98 (71.5)	144 (77.8)	< 0.001
Previous stroke	11 (2.3)	4 (2.9)	9 (4.9)	0.19
Dyslipidemia	156 (33.2)	51 (37.2)	83 (44.9)	0.074
Chronic kidney disease	214 (45.5)†	84 (61.3)	119 (64.3)	<0.001
Atrial fibrillation	100 (21.3)	33 (24.1)	48 (25.9)	0.723
Smoking	38 (8.1)	6 (4.4)	14 (7.6)	0.68
Hemodialysis	19 (4.0)	11 (8.0)	7 (3.8)	0.309
LVEF, %	58.16 ± 14.73	59.73 ± 14.85	57.87 ± 15.56	0.492
NYHA class				0.251
I	206 (43.8)	49 (35.8)	91 (49.2)	
II	212 (45.1)	71 (51.8)	80 (43.2)	
III	45 (9.6)	16 (11.7)	13 (7.0)	
IV	7 (1.5)	1 (0.7)	1 (0.5)	
MNA-SF	12.03 ± 2.13∗^,^†	11.54 ± 2.30†	10.52 ± 2.44	< 0.001
Type of surgery				< 0.001
CABG	48 (10.2)†	9 (6.6)†	30 (16.2)	
Valve	251 (53.4)	71 (51.8)	63 (34.1)	
Concomitant	15 (3.2)	6 (4.4)	12 (6.5)	
Aortic	156 (33.2)	51 (37.2)	80 (43.2)	
EuroSCORE II	5.70 ± 2.41∗^,^†	6.85 ± 2.44	7.14 ± 2.25	< 0.001
Medications				
β-blockers	217 (46.2)	64 (46.7)	80 (43.2)	0.799
ACE-I	105 (22.3)	38 (27.7)	41 (22.2)	0.715
ARB	225 (47.9)	72 (52.6)	100 (54.1)	0.285
Statin	172 (36.6)	57 (41.6)	90 (48.6)	0.018
Diuretics	177 (37.7)	62 (45.3)	71 (38.4)	0.517
Physical function				
Gait speed, m/s	0.94 ± 0.23∗^,^†	1.00 ± 0.21	1.06 ± 0.22	< 0.001
Handgrip, kg	29.59 ± 9.13∗^,^†	25.37 ± 7.36	22.84 ± 8.36	< 0.001
SPPB, points	11.26 ± 1.40∗^,^†	10.69 ± 1.73	10.27 ± 1.96	< 0.001
Sarcopenia	138 (29.4)†	50 (36.5)	72 (38.9)	0.039

Data are presented as mean ± standard deviation or n (%).

ACE-I, angiotensin-converting enzyme inhibitor; ARB, angiotensin II receptor blocker; BMI, body mass index; BNP, brain natriuretic peptide; CABG, coronary artery bypass grafting; COPD, chronic obstructive pulmonary disease; CRP, C-reactive protein; eGFR, estimated glomerular filtration rate; EuroSCORE II, European System for Cardiac Operative Risk Evaluation; LVEF, left ventricular ejection fraction; MNA-SF, Mini Nutritional Assessment-Short Form; NYHA, New York Heart Association; SPPB, Short Physical Performance Battery.

∗ Significant difference compared with number of teeth 10-19.

† Significant difference compared with number of teeth < 10.

**Table 2:** Baseline oral health status

Variable	Total	Number of teeth			*P*
		≥ 20	10-19	< 10	
Number	792	470	137	185	
Remaining teeth, n	18.69 ± 9.87	25.85 ± 2.89∗^,^†	14.98 ± 2.98†	3.25 ± 3.26	< 0.001
Dentures	276 (34.8)	57 (12.1)∗^,^†	67 (48.9)†	152 (82.2)	< 0.001
CPITN, points	2.20 ± 1.21	2.34 ± 1.01∗^,^†	2.66 ± 1.09†	1.46 ± 1.18	< 0.001
No. of CPITN ≥4	137 (17.3)	68 (14.5)∗	44 (32.4) †	25 (13.6)	0.001
DMF-T					
D-T	0.43 ± 1.35	0.47 ± 1.37†	0.68 ± 1.89†	0.14 ± 0.59	0.001
M-T	13.33 ± 9.86	6.18 ± 2.88∗^,^†	17.02 ± 2.98†	28.76 ± 3.26	< 0.001
F-T	7.52 ± 5.73	9.52 ± 5.61∗^,^†	8.24 ± 4.07†	1.90 ± 2.54	< 0.001
Eichner index					< 0.001
Class A	311 (39.3)	308 (65.5)∗^,^†	3 (2.2)†	0 (0.0)	
Class B	282 (35.6)	162 (34.5)	108 (78.8)	12 (6.5)	
Class C	199 (25.1)	0 (0.0)	26 (19.0)	173 (93.5)	
Prophylactic dental extractions before surgery	96 (12.1)	43 (9.1)∗	32 (23.4)†	21 (11.4)	< 0.001

Data are presented as mean ± standard deviation or n (%).

CPITN, Community Periodontal Index of Treatment Needs; DMF-T; decayed, missing, and filled teeth.

∗ Significant difference compared with number of teeth 10-19.

† Significant difference compared with number of teeth < 10.

### Postoperative complications and functional recovery

Hospital mortality was significantly different based on the number of remaining teeth; however, the difference was not statistically significant in the analysis of variance post-hoc analysis (Table 3). The prevalence of postoperative delirium was significantly higher in patients with fewer teeth (*P* = 0.004). The incidences of postoperative pneumonia and reintubation were significantly higher in patients with <10 remaining teeth than in patients with ≥ 20 teeth (*P* < 0.05 for each). Nevertheless, the association between postoperative pneumonia or reintubation and the number of remaining teeth was not statistically significant after adjusting for other confounding variables in multivariable logistic regression analysis (*P* = 0.07 and *P* = 0.08, respectively). The results of the multiple regression analysis for predicting the progress of postoperative rehabilitation are shown in Table 4. After adjusting for all confounding factors, the number of teeth remained a statistically significant predictor of functional recovery, that is, length of hospital stay, days to postoperative independent walking, and Barthel index after surgery (*P* < 0.05: adjusted R^2^ = 0.36, 0.24, and 0.34, respectively).

**Table 3:** Postoperative course according to number of remaining teeth

Variable	Number of teeth			*P*
	≥ 20	10-19	< 10	
Number	470	137	185	
Hospital mortality	6 (1.3)	0 (0.0)	6 (3.2)	0.040
Postoperative AF	209 (44.5)	58 (42.3)	97 (52.4)	0.488
Sternal wound infection	8 (1.7)	2 (1.5)	3 (1.6)	0.780
Delirium	74 (15.7)∗^,^†	31 (22.6)†	52 (28.1)	0.004
Stroke	25 (5.3)	10 (7.3)	17 (9.2)	0.544
Pneumonia	8 (1.7)†	6 (4.4)	11 (5.9)	0.015
Tracheostomy	4 (0.9)	3 (2.2)	7 (3.8)	0.115
Reintubation	4 (0.9)†	5 (3.6)	11 (5.9)	0.012
Discharge status				< 0.001
Home	423 (90.0)∗^,^†	110 (80.3)†	125 (67.6)	
Transfer	41 (8.7)	27 (19.7)	54 (29.2)	
ICU stay, d	2.08 ± 1.54	2.55 ± 3.38	3.08 ± 5.23	0.078
Gait independent, d	4.48 ± 3.62∗^,^†	6.94 ± 4.26	7.31 ± 4.89	0.002
Barthel index, points	96.24 ± 9.05∗^,^†	93.27 ± 13.57	92.59 ± 13.29	0.042
Hospital stay, d	20.67 ± 11.43∗^,^†	26.37 ± 11.07†	28.27 ± 15.13	0.005

Data are expressed as mean ± standard deviation or n (%).

AF, atrial fibrillation; ICU, intensive care unit.

∗ Significant difference compared with number of teeth 10-19.

† Significant difference compared with number of teeth < 10.

**Table 4:** Multivariate regression analysis of functional recovery after surgery

Variable	Independent walking R^2^ = 0.24 Adjusted R^2^ = 0.20 *P* < 0.0001 F = 5.49		Barthel index R^2^ = 0.34 Adjusted R^2^ = 0.31 *P* < 0.0001 F = 14.34		Length of hospital stay R^2^ = 0.36 Adjusted R^2^ = 0.32 *P* < 0.0001 F = 8.90		
	Standardized β (95% CI)	*P*	Standardized β (95% CI)	*P*		Standardized β (95% CI)	*P*
Age	–0.05 (–0.15 - 0.04)	0.33	–0.11 (–0.23 - –0.02)	0.02		0.05 (–0.18 - 0.64)	0.27
Sex, female	–0.07 (–1.05 - 0.35)	0.33	–0.06 (–1.97 - 0.71)	0.36		–0.01 (–5.63 - 4.76)	0.87
BMI	–0.08 (–0.26 - 0.07)	0.24	0.18 (–0.11 - 0.38)	0.11		0.09 (–0.16 - 1.93)	0.57
MNA-SF	–0.12 (–0.43 - 0.06)	0.08	0.13 (0.05 - 1.16)	0.03		–0.09 (–4.18 - –0.05)	0.02
SPPB	–0.32 (–1.50 - –0.19)	<0.001	0.47 (0.26 - 3.71)	< 0.001		–0.10 (–6.78 - –0.05)	0.01
EuroSCORE II	0.10 (–0.03 - 0.30)	0.11	0.01 (–0.29 - 0.35)	0.86		–0.03 (–1.43 - 0.56)	0.39
Number of teeth	–0.11 (–1.1 - –0.10)	0.02	0.18 (0.04 - 0.20)	0.01		–0.28 (–1.54 - –0.17)	0.01
Postoperative pneumonia	0.13 (–1.09 - 2.00)	0.51	–0.24 (–5.35 - –0.13)	0.02		0.35 (0.15 - 1.15)	0.01
Type of surgery	0.11 (–1.20 - 3.45)	0.78	–0.05 (–5.57 - 3.47)	0.65		–0.07 (–12.14 - 3.81)	0.31
NYHA class	0.05 (–0.18 - 1.44)	0.13	–0.08 (–3.16 - –0.07)	0.03		0.11 (0.05 - 14.21)	0.02
Chronic kidney disease	0.02 (–0.56 - 0.73)	0.79	0.04 (–0.84 - 1.63)	0.33		0.08 (–0.15 - 9.21)	0.10
Smoking	0.04 (–0.47 - 0.88)	0.55	–0.04 (–1.73 - 0.81)	0.48		0.01 (–4.56 - 5.49)	0.86

Data are presented as mean ± standard deviation, or n (%).

BMI, body mass index; CI, confidence interval; EuroSCORE, European System for Cardiac Operative Risk Evaluation; MNA-SF, Mini Nutritional Assessment—Short Form; NYHA, New York Heart Association; SPPB, Short Physical Performance Battery.

## Discussion

To the best of our knowledge, this is the first study to demonstrate the relationship between oral health status and postoperative outcomes in patients after cardiovascular surgery. The incidence of postoperative respiratory complications was significantly higher in patients with severe tooth loss. Furthermore, the number of remaining teeth was independently associated with functional recovery after adjustment for other confounding variables.

The oral cavity is an important part of human health, and the number of remaining teeth represents comprehensive oral function. Healthy older individuals with > 20 teeth reportedly maintained several oral function criteria across the aging process, including oral mucosal wetness, occlusal force, lip motor function, masticatory function, and swallowing function.^26^ Thus, we focused on the number of teeth as a simple, easy-to-understand, and clinically applicable index of oral health. In the present study, 59.3% of patients had ≥ 20 teeth, which is substantially lower than the number in age-matched healthy populations (73.0%).^27^ There may be several explanations for this difference. First, tooth loss reflects an accumulation of oral inflammation and contributes to CVD. Enhancement of inflammation mediators such as cytokines triggers periodontal disease that may spread systemically and is involved in the onset and progression of cardiometabolic disorders.^28^ Moreover, periodontal disease is a cause of tooth loss.^29^ Second, a high prevalence of smoking causes poor oral health status and CVD risk.^30^ Although all patients were instructed to stop smoking before cardiovascular surgery, smoking rates remained high in this study. Third, patients with increased tooth loss may have attenuated mastication, which could lead to poorer nutrient intake. Oral dysfunction is associated with inadequate nutrient intake, which increases the risk of CVD.^31^, ^32^, ^33^ Fourth, diabetes is a well-known major risk factor for CVD.^34^ Moreover, Demmer et al. showed that periodontitis and diabetes could be bidirectional, and periodontitis severity was associated with diabetes risk.^35^ In our cohort, having fewer remaining teeth (<10) was associated with incident diabetes more than having >10 teeth, consistent with the previous study. It is plausible that poor oral health is more common, collectively, in cardiovascular surgery patients, and we demonstrated this relationship for the first time in a large cohort.

Oral health status was an independent predictor of functional recovery after cardiovascular surgery. Among many assessment tools of oral health status, the number of teeth was the most sensitive for predicting the progress of rehabilitation. Nutritional status may mediate between oral function and functional recovery. We previously reported that preoperative nutritional status or dietary intake was an independent predictor of rehabilitation progress after cardiac surgery.^9^^,^^36^ Perioperative low-protein intake was associated with increasing catabolism and inhibiting anabolism of muscle mass, which slowed functional recovery. Highly invasive surgery, such as cardiopulmonary bypass, results in muscle catabolism that greatly enhances whole-body protein loss, indicating increased breakdown of muscle proteins, particularly myofibrillar proteins.^37^ Preoperative malnutrition and insufficient postoperative dietary intake, due to oral problems, are highly suggestive of a prolongation of the catabolic reaction and further decline in nutritional status. Systemic inflammatory processes caused by poor oral health may enhance the catabolic reaction. Additionally, poor oral health is associated with many chronic illnesses, such as diabetes and CKD, that cause a decrease in the functional reserve capacity, leading to prolonged functional recovery.^24^

Physical function was also an important predictor of postoperative rehabilitation progress. Here, individuals with ≥20 teeth had significantly better physical function than those with <20 teeth. Evidence to demonstrate the relationship between oral health status and physical function in CVD patients has been insufficient; nevertheless, a large cohort study revealed that a low number of natural teeth was associated with physical frailty or mortality in community-dwelling older individuals.^38^ Our findings confirmed an association between oral health status and physical function, even in cardiovascular surgery patients. Moreover, after adjusting for physical function, the number of teeth remained an independent predictor of functional recovery. Oral status might be predictive of frailty development and postoperative rehabilitation progress after cardiovascular surgery. Thus, the number of remaining teeth may be used to screen for physical frailty.

The incidence of postoperative pneumonia was higher in patients with <10 remaining teeth. The oral cavity may be an important reservoir of bacteria responsible for causing lung infection, potentially leading to ventilator-associated pneumonia.^39^ Bergan et al. demonstrated that poor oral health status increased the risk of postoperative pneumonia after cardiac surgery.^40^ In recent years, preoperative oral intervention has received considerable attention for its effectiveness in preventing nosocomial pneumonia in cardiovascular surgery patients.^10^^,^^40^^,^^41^ Suzuki et al. revealed that preoperative periodontal treatment can reduce the risk of postoperative infection.^42^ On the other hand, a recent systematic review demonstrated that it is unclear whether dental treatment before cardiovascular surgery prevents postoperative complications or not.^43^ Although the details of the specific interventions and timing are controversial, early detection of oral problems to facilitate and evaluate oral care procedures is required to prevent postoperative respiratory complications and facilitate functional recovery in older patients. In clinical practice, careful attention must be paid to oral health conditions, and a strategy for improving oral hygiene practices, promoting the retention of natural teeth, and preoperative oral care is critical. Older frail patients who are at high risk of surgery and decreased reserve capacity would benefit most from oral examination and preoperative intervention.^7^ A nationwide survey in Japan reported that CVD patient care was provided by various staff members and doctors.^44^ A multidisciplinary team approach is superior to standard care in cardiac surgery patients to reduce the risk of readmission,^45^ consistent with our findings. Further studies are required to elucidate the effect of a multidisciplinary approach on postoperative rehabilitation, long-term prognosis, and patient-reported outcomes.

There are several limitations to this study. First, this was a retrospective single-centre cohort study with a limited sample size. Generalizability was limited because of selection bias; however, multiple regression analysis was used to adjust for baseline characteristics and confirm the results. Second, the follow-up period was the duration of the hospital stay. Thus, the effect of oral health status on long-term prognosis and patient-reported outcomes cannot be discussed. Third, because of the retrospective design, details of oral health status, including swallowing function, oral diadochokinesis, and mastication ability, were unavailable. Masticatory muscle strength and power could be correlated with whole-body muscle strength or muscle mass. Fourth, oral health can be attributed to various factors, and there are unknown confounding factors that have not been investigated in this study, such as economic status and patients’ health literacy. Thus, further interventional studies for oral health are needed to clarify the effect of oral status on patient outcomes. Despite these limitations, this is the largest study to demonstrate the effects of preoperative oral health status on postoperative outcomes and include a comprehensive assessment of oral health. The results of the present study are unique and pave the way for future clinical studies for multidisciplinary professionals.

## Conclusions

Oral health status deteriorated in patients with severe tooth loss compared with an age-matched population. The incidence of postoperative pneumonia was higher in patients with severe tooth loss. Moreover, preoperative oral health status was independently associated with functional recovery. Our findings demonstrate the importance of evaluating preoperative oral health status for postoperative outcomes. The clinical effects of oral health status on long-term outcomes and prognosis should be investigated in the future.

## Funding Sources

This study was supported by the Hyogo Physical Therapist Association and the Japanese Association of Cardiac Rehabilitation. This work was supported by a grant from JSPS KAKENHI (No. JP20K19447).

## Disclosures

The other authors have no conflicts of interest to disclose.

## References

[1] Hanne K., Ingelise T., Linda C., Ulrich P.P. (2012). Oral status and the need for oral health care among patients hospitalised with acute medical conditions. J Clin Nurs.

[2] Sanchez P., Everett B., Salamonson Y. (2019). The oral health status, behaviours and knowledge of patients with cardiovascular disease in Sydney Australia: a cross-sectional survey. BMC Oral Health.

[3] Rassameehiran S., Klomjit S., Mankongpaisarnrung C., Rakvit A. (2015). Postextubation dysphagia. Proc (Bayl Univ Med Cent).

[4] Chen C.C., Wu K.H., Ku S.C. (2018). Bedside screen for oral cavity structure, salivary flow, and vocal production over the 14 days following endotracheal extubation. J Crit Care.

[5] Minakuchi S., Tsuga K., Ikebe K. (2018). Oral hypofunction in the older population: Position paper of the Japanese Society of Gerodontology in 2016. Gerodontology.

[6] Rapp L., Sourdet S., Vellas B., Lacoste-Ferré M.H. (2017). Oral health and the frail elderly. J Frailty Aging.

[7] Lin H.S., Watts J.N., Peel N.M., Hubbard R.E. (2016). Frailty and postoperative outcomes in older surgical patients: a systematic review. BMC Geriatr.

[8] Yuguchi S., Saitoh M., Oura K. (2019). Impact of preoperative frailty on regaining walking ability in patients after cardiac surgery: multicenter cohort study in Japan. Arch Gerontol Geriatr.

[9] Ogawa M., Izawa K.P., Satomi-Kobayashi S. (2017). Poor preoperative nutritional status is an important predictor of the retardation of rehabilitation after cardiac surgery in elderly cardiac patients. Aging Clin Exp Res.

[10] Akashi M., Nanba N., Kusumoto J., Komori T. (2019). Perioperative intervention by oral medicine team in cardiovascular surgery patients. Gen Thorac Cardiovasc Surg.

[11] Ogawa M., Izawa K.P., Satomi-Kobayashi S. (2017). Impact of delirium on postoperative frailty and long term cardiovascular events after cardiac surgery. PLoS One.

[12] Takahashi T., Kumamaru M., Jenkins S. (2015). In-patient step count predicts re-hospitalization after cardiac surgery. J Cardiol.

[13] Afilalo J., Eisenberg M.J., Morin J.F. (2010). Gait speed as an incremental predictor of mortality and major morbidity in elderly patients undergoing cardiac surgery. J Am Coll Cardiol.

[14] Guigoz Y. (2006). The Mini Nutritional Assessment (MNA) review of the literature—what does it tell us?. J Nutr Health Aging.

[15] Guralnik J.M., Simonsick E.M., Ferrucci L. (1994). A short physical performance battery assessing lower extremity function: association with self-reported disability and prediction of mortality and nursing home admission. J Gerontol.

[16] Hamaguchi Y., Kaido T., Okumura S. (2016). Proposal for new diagnostic criteria for low skeletal muscle mass based on computed tomography imaging in Asian adults. Nutrition.

[17] Flanagan P.G., Findlay G.P., Magee J.T. (2000). The diagnosis of ventilator-associated pneumonia using non-bronchoscopic, non-directed lung lavages. Intensive Care Med.

[18] World Health Organization (1997). World Health Organization Oral Health Surveys – Basic Methods.

[19] Eichner K. (1990). Renewed examination of the group classification of partially edentulous arches by Eichner and application advices for studies on morbidity statistics. Stomatol DDR.

[20] Ainamo J., Barmes D., Beagrie G. (1982). Development of the World Health Organization (WHO) community periodontal index of treatment needs (CPITN). Int Dent J.

[21] JCS Joint Working Group (2014). Guidelines for rehabilitation in patients with cardiovascular disease (JCS 2012). Circ J.

[22] Arai Y., Kimura T., Takahashi Y. (2019). Preoperative frailty is associated with progression of postoperative cardiac rehabilitation in patients undergoing cardiovascular surgery. Gen Thorac Cardiovasc Surg.

[23] Mahoney F.I., Barthel D.W. (1965). Functional evaluation: the Barthel Index. Md State Med J.

[24] Shin H.S. (2018). Number of existing permanent teeth is associated with chronic kidney disease in the elderly Korean population. Korean J Intern Med.

[25] Hayasaka K., Tomata Y., Aida J. (2013). Tooth loss and mortality in elderly Japanese adults: effect of oral care. J Am Geriatr Soc.

[26] Iyota K., Mizutani S., Oku S. (2020). A cross-sectional study of age-related changes in oral function in healthy Japanese individuals. Int J Environ Res Public Health.

[27] Japanese Ministry of Health Labour and Welfare Results of the 2016 survey on dental diseases (summary). https://www.mhlw.go.jp/toukei/list/dl/62-28-02.pdf.

[28] De Oliveira C., Watt R., Toothbrushing Hamer M. (2010). inflammation, and risk of cardiovascular disease: results from Scottish Health Survey. BMJ.

[29] Joshipura K.J., Hung H.C., Rimm E.B., Willett W.C., Ascherio A. (2003). Periodontal disease, tooth loss, and incidence of ischemic stroke. Stroke.

[30] Hanioka T., Ojima M., Tanaka K. (2011). Causal assessment of smoking and tooth loss: a systematic review of observational studies. BMC Public Health.

[31] Gaewkhiew P., Sabbah W., Bernabe E. (2017). Does tooth loss affect dietary intake and nutritional status? A systematic review of longitudinal studies. J Dent.

[32] Hung H.C., Colditz G., Joshipura K.J. (2005). The association between tooth loss and the self-reported intake of selected CVD-related nutrients and foods among US women. Community Dent Oral Epidemiol.

[33] Hutton B., Feine J., Morais J. (2002). Is there an association between edentulism and nutritional state?. J Can Dent Assoc.

[34] Kato M., Noda M., Mizoue T. (2015). Diagnosed diabetes and premature death among middle-aged Japanese: results from a large-scale population-based cohort study in Japan (JPHC study). BMJ Open.

[35] Demmer R.T., Jacobs D.R., Desvarieux M. (2008). Periodontal disease and incident type 2 diabetes: results from the First National Health and Nutrition Examination Survey and its epidemiologic follow-up study. Diabetes Care.

[36] Ogawa M., Izawa K.P., Satomi-Kobayashi S. (2019). Effects of postoperative dietary intake on functional recovery of patients undergoing cardiac surgery. Nutr Metab Cardiovasc Dis.

[37] Wray C.J., Mammen J.M., Hasselgren P.O. (2002). Catabolic response to stress and potential benefits of nutrition support. Nutrition.

[38] Aida J., Kondo K., Hirai H. (2012). Association between dental status and incident disability in an older Japanese population. J Am Geriatr Soc.

[39] Munro C.L., Grap M.J., Elswick R.K. (2006). Oral health status and development of ventilator-associated pneumonia: a descriptive study. Am J Crit Care.

[40] Bergan E.H., Tura B.R., Lamas C.C. (2014). Impact of improvement in preoperative oral health on nosocomial pneumonia in a group of cardiac surgery patients: a single arm prospective intervention study. Intensive Care Med.

[41] Starks B., Harbert C. (2011). Aspiration prevention protocol: decreasing postoperative pneumonia in heart surgery patients. Crit Care Nurse.

[42] Suzuki H., Matsuo K., Okamoto M. (2019). Preoperative periodontal treatment and its effects on postoperative infection in cardiac valve surgery. Clin Exp Dent Res.

[43] Lockhart P.B., DeLong H.R., Lipman R.D. (2019). Effect of dental treatment before cardiac valve surgery: systematic review and meta-analysis. J Am Dent Assoc.

[44] Kamiya K., Yamamoto T., Tsuchihashi-Makaya M. (2019). Nationwide survey of multidisciplinary care and cardiac rehabilitation for patients with heart failure in Japan—an analysis of the AMED-CHF study. Circ J.

[45] Ogawa M., Satomi-Kobayashi S., Yoshida N. Effects of acute-phase multidisciplinary rehabilitation on unplanned readmissions after cardiac surgery [e-pub ahead of print]. J Thorac Cardiovasc Surg. 10.1016/j.jtcvs.2019.11.069.

